# The Role of Vitamin D in Combination Treatment for Patients With Rheumatoid Arthritis

**DOI:** 10.3389/fmed.2020.00312

**Published:** 2020-07-15

**Authors:** Jianhong Wu, Jianling Dong, Shilin Li, Jiaang Luo, Yu Zhang, Hong Liu, Yuanpiao Ni, Xue Li, Jun Zhou, Hang Yang, Qianrong Xie, Xuejun Jiang, Tingting Wang, Pingxi Wang, Fanwei Zeng, Yanpeng Chu, Jing Yang, Fanxin Zeng

**Affiliations:** ^1^Department of Rheumatology, Dazhou Central Hospital, Dazhou, China; ^2^Department of Rheumatology, Mianyang Central Hospital, Mianyang, China; ^3^Department of Clinical Research Center, Dazhou Central Hospital, Dazhou, China

**Keywords:** rheumatoid arthritis, vitamin D, conventional synthetic disease-modifying antirheumatic drugs, response to treatment, disease activity

## Abstract

**Background:** The aim of this study is to evaluate the clinical efficacy of vitamin D (VitD) supplementation in terms of response to treatment and improvement of disease activity in rheumatoid arthritis (RA).

**Methods:** This study analyzed 1180 RA patients' records treated at Mianyang Central Hospital from February 2015 to July 2019. The patients were allocated into VitD group and control group based on their medical regimens. The outcome measures were primary efficacy, defined as treatment response-based EULAR response criteria in RA, and secondary efficacy, defined as improvement in disease activity indicators. Safety was evaluated according to the incidence of all-cause infections.

**Results:** At month 6, the primary efficacy revealed that there were 22.8% good responders and 19.0% moderate responders in the VitD group, and 22.3% good responders and 22.3% moderate responders in the control group; there were no differences between the two groups (*p* = 0.754). The similar primary efficacy outcomes were observed at months 3, 12, and >12. The secondary efficacy indicated that there were no differences in most indexes between the two groups at months 1, 3, 6, 12, and >12. The subgroups (based on baseline DAS28 (CRP), glucocorticoids use and disease duration) analysis results suggested that VitD group didn't have the advantage for treating RA. The incidence of infections was similar in the two groups.

**Conclusion:** VitD supplementation did not provide additional benefit for anti-rheumatic treatment. These data supported the need for prospective, randomized, controlled trials to evaluate the role of VitD supplementation in treating RA.

## Introduction

Rheumatoid arthritis (RA) is a progressive and chronic inflammatory joint disease with cartilage and bone damage that leads to disability (1). RA has an incidence of ~0.5% worldwide for adults (2). Environmental factors, genetics and female sex are known to be an important risk factor for RA (3). Although the treatment of RA diseases has improved, mainly using glucocorticoids, anti-inflammatory drugs, conventional synthetic disease-modifying antirheumatic drugs (csDMARDs), and targeted synthetic DMARDs (4), some patients still do not respond to these treatments. Therefore, it is important to investigate the reason for patient intolerance and improve current therapies or discover new treatment options.

Vitamin D (VitD) deficiency, as an environmental risk factor, is significantly associated with high disease activity and neuropathic pain in RA patients (5–7). The proportion of VitD deficiency was 43% in RA patients (8). Although the mechanism of the VitD effect on RA is incompletely understood, VitD may play a potential role in the occurrence and development of RA by decreasing the production of proinflammatory cytokines, including tumor necrosis factor-α, interleukin-1, interleukin-6, and interleukin-17 (9). VitD levels are inversely associated with the RA disease activity (10–12) and VitD has an immunoregulatory function as well (9), so VitD supplementation is considered to have potential therapeutic benefits for RA. At present, relatively few randomized clinical trials have assessed VitD supplementation in RA (13–15). An exploratory study suggested that VitD supplementation improved 28 joint disease activity scores based on C-reactive protein (DAS28 (CRP)) in patients without VitD deficiency but did not affect patients with VitD deficiency at baseline (14). Another study reported that VitD supplementation for more than 3 months could significantly improve disease activity in patients with persistent disease activity and VitD deficiency (16). Additionally, there was a tendency of reduced recurrence in RA patients after VitD supplementation (17). However, the clinical efficacy of VitD supplementation on the response to treatment of disease activity in RA remains unclear.

The aim of this study was to investigate the anti-rheumatic effect of VitD supplementation as indicated by response to treatment and improvement of disease activity in RA patients.

## Patients and Methods

### Study Population

**Figure 1:**
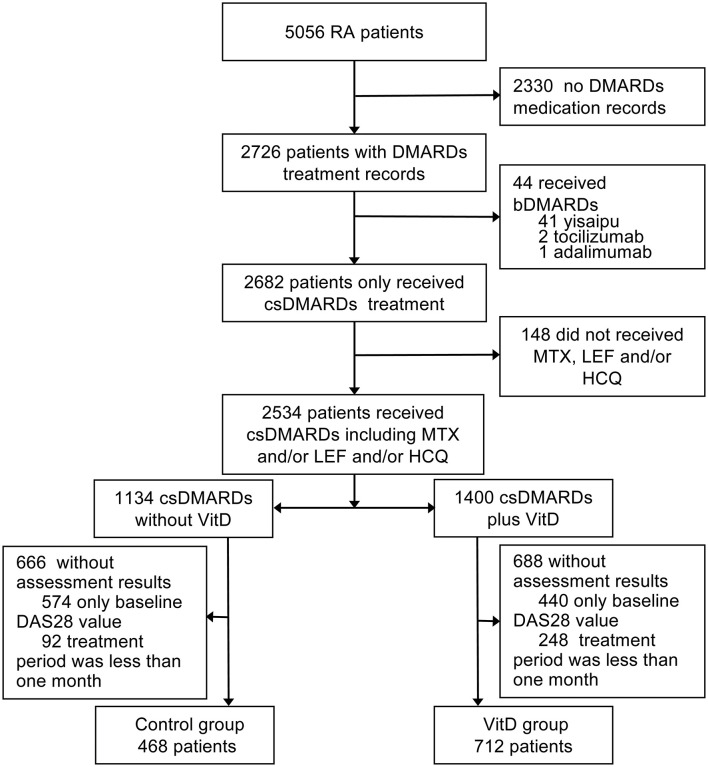
Study profile. RA, rheumatoid arthritis; bDMARDs, biological disease modifying antirheumatic drugs; csDMARDs, conventional synthetic disease modifying antirheumatic drugs; DMARDs, disease modifying antirheumatic drugs; MTX, methotrexate; LEF, leflunomide; HCQ, hydroxychloroquine; DAS28, 28 joint disease activity scores; VitD, vitamin D. Yisaipu, a recombinant human TNFR:Fc protein, is a biosimilar of etanercept widely used in China.

This retrospective study was approved by the ethics committees and institutional review boards of Dazhou Central Hospital, and the ethics committee waived the need for patients to sign informed consent. The total medical records of 5056 RA patients from Mianyang Central Hospital were analyzed between February 2015 and July 2019. All patients were confirmed to have RA according to the 1987 ACR classification criteria and/or the 2010 revised American College of Rheumatology/European League Against Rheumatism classification criteria (18), were age ≥ 18 years and were on continued treatment with csDMARDs. We excluded patients with missing medication information; patients who received any biologics (yisaipu (biosimilar of etanercept) (19), tocilizumab, adalimumab) for RA treatment; patients who did not receive any methotrexate (MTX), leflunomide (LEF) or hydroxychloroquine (HCQ); patients without posttreatment disease activity score assessments; and patients with a treatment period <1 month (Figure 1). The included patients were divided into two groups: the VitD group: patients treated with csDMARDs combined with VitD; and the control group: patients treated with csDMARDs who never received VitD. The baseline time was defined as the first day of VitD supplementation in the VitD group and the date of first assessment of DAS28 (CRP) from medical records in the control group.

In the study cohort, 10 to 20 mg/week MTX, 10 mg/day LEF, and 400 mg/day HCQ monotherapy or combination therapy were administered.

### VitD Supplementation

The duration of VitD supplementation was the accumulation of days (may not be continuous) during RA treatment. The average duration of VitD supplementation was 4.4 ± 4.9 months. VitD was used orally for patients and included calcitriol (0.25 to 0.5 μg/day), or calcium carbonate and vitamin D3 tablets (200 to 400 IU/day).

### Clinical Efficacy

The primary efficacy endpoint was defined as the percentage of patients with moderate or good response to RA treatment at months 3, 6, 12, and >12. It was assessed and determined based on the EULAR response criteria (20, 21) in RA (good response, ΔDAS28 > 1.2 and final DAS28 ≤ 3.2; moderate response, ΔDAS28 > 0.6 and ≤ 1.2 and final DAS28 ≤ 5.1 or ΔDAS28 > 1.2 and final DAS28 > 3.2; non-response, ΔDAS28 ≤ 0.6 or ΔDAS28 > 0.6 and ≤ 1.2 and final DAS28 > 5.1; ΔDAS28: mean improvement from baseline).

The secondary efficacy endpoint was defined as the assessed improvement in disease activity at month 1, 3, 6, 12, and > 12. The assessment indicators included DAS28 (CRP), the health assessment questionnaire (HAQ) (22), morning stiffness, the health survey summary (23), C-reaction protein (CRP), erythrocyte sedimentation rate (ESR), the patient global assessment (PtGA), tender 28-joint counts (TJC28), and swollen 28-joint counts (SJC28).

### Adverse Events

Safety was primarily evaluated according to the proportion of infections, including lung infection, bronchiectasis with infection, interstitial lung disease with infection, urinary tract infection, infectious fever, and anterior gastric ulcer with Helicobacter pylori infection, in both the VitD and control groups.

### Statistical Analysis

All statistical analyses were performed using SPSS version 20.0 (IBM, Armonk, NY, USA). The variable means between groups were compared by the independent samples *t-*test and one-way analysis of variance, followed by LSD's test. All means are reported with the corresponding standard deviation in the tables, and *p* < 0.05 were regarded as statistically significant. Categorical variables were compared by the Chi-square test. All results were exclusively analyzed based on available data, and no assumptions were made for missing data.

## Results

Out of the 5056 RA patients screened, 1,180 patients fulfilled the inclusion criteria for analysis. The baseline characteristics of the two groups are presented in Table 1. The VitD group included 712 patients (age, 52.19 ± 11.86 years; 82.7% women). The control group included 468 patients (age, 52.42 ± 12.00 years; 80.1% women). The mean disease duration in the VitD group was significantly less than control group (75.89 ± 82.61 vs. 85.06 ± 90.90 months, *p* = 0.009).

**Table 1:** Demographics and disease baseline characteristics.

**Characteristics**	**VitD group (*N* = 712)**	**Control group (*N* = 468)**	***P***
Age (years)	52.19 ± 11.86	52.42 ± 12.00	0.472
≤ 50	304 (42.7)	210 (44.9)	
> 50	408 (57.3)	258 (55.1)	
Sex			0.281
Female (%)	589 (82.7)	375 (80.1)	
Male (%)	123 (17.3)	93 (19.9)	
Disease duration (months)	75.89 ± 82.61	85.06 ± 90.90	0.009^**^
≤ 24	127 (17.8)	46 (9.8)	
> 24 and ≤ 60	307 (43.1)	175 (37.4)	
> 60	233 (32.7)	155 (33.1)	
Miss data	45 (6.3)	92 (19.7)	
Disease activity			
DAS28 (CRP)	3.24 ± 1.42	3.64 ± 1.51	<0.001^***^
≤ 3.2	404 (56.7)	214 (45.7)	
> 3.2	308 (43.3)	254 (54.3)	
HAQ	1.38 ± 2.83	2.36 ± 3.57	<0.001^***^
Morning stiffness (minutes)	9.52 ± 22.06	19.70 ± 36.09	<0.001^***^
Health survey summary	70.31 ± 17.35	66.54 ± 19.52	0.014^*^
DAS28 parameters			
TJC28	6.20 ± 6.78	7.54 ± 7.65	0.012^*^
SJC28	5.54 ± 5.87	6.09 ± 6.55	0.315
ESR (mm/h)	22.08 ± 27.75	19.64 ± 27.33	0.172
CRP (mg/L)	11.95 ± 22.19	15.59 ± 23.64	0.012^*^
PtGA	44.23 ± 18.42	46.44 ± 20.98	0.073
Duration of VitD supplement	4.4 ± 4.9		
1–3 months	455 (63.9)	————	
4–6 months	104 (14.6)	————	
> 6 months	152 (21.3)	————	
Oral GCs			<0.001^***^
GCs present	223 (31.3)	101 (21.6)	
GCs absent	482 (67.7)	367 (78.4)	
Use of csDMARDs			0.439
Methotrexate	507 (71.2%)	301 (64.3%)	
Leflunomide	639 (89.7%)	424 (90.6%)	
Hydroxychloroquine	455 (63.9%)	274 (58.5%)	

*All values are n (%), mean ± SD. DAS 28, 28-joint disease activity score; TJC28, tender 28-joint counts; SJC28, swollen 28-joint counts; ESR, erythrocyte sedimentation rate; CRP, C-reaction protein; PtGA, patient global assessment; HAQ, health assessment questionnaire; GCs, glucocorticoids; csDMARDs, conventional synthetic disease modifying antirheumatic drugs*.

* *p < 0.05*;

** *p < 0.01*;

*** *p < 0.001*.

Patients in the VitD group had significantly lower baseline disease activity, including DAS28 (CRP), HAQ, morning stiffness, compared with control group. The statistical differences were found in TJC-28 and CRP between the two groups at baseline, (*p* = 0.012; *p* = 0.012, respectively). The proportion of patients with baseline DAS28 (CRP) ≤ 3.2 was 56.7% (404/712) in the VitD group and 45.6% (214/468) in the control group.

### Clinical Efficacy

The primary outcome was the DAS-28-based EULAR response level for the two cohorts (Table 2). At month 3, there were 21.6% good response cases and 20.0% moderate for the VitD group, 21.4% good response cases and 20.9% moderate for the control group, without differences significantly (*p* = 0.937). Subsequently, we analyzed the outcome at month 6, the response level was good in 22.8% of patients and moderate in 19.0% for the VitD group. For the control group, good response was achieved in 22.3% of cases and moderate in 22.3% failed to respond. Response to antirheumatic treatment did not differ significantly between the two cohorts (*p* = 0.754). The analysis revealed a similar result at month 12, good in 19.5% and moderate in 17.6 for the VitD group, good in 20.1% and moderate in 18.8 for the control group (*p* = 0.499).

**Table 2:** DAS-28-based EULAR response in patients at various timepoints (months 3, 6, 12, and >12).

**Timepoints**	**DAS28-based EULAR response**	**VitD group**	**Control group**	***P***
Month 3	Good response	82 (21.6)	39 (21.4)	0.937
	Moderate response	76 (20.0)	38 (20.9)	
	Non-response	222 (58.4)	105 (57.7)	
Month 6	Good response	86 (22.8)	43 (22.3)	0.754
	Moderate response	72 (19.0)	43 (22.3)	
	Non-response	220 (58.2)	107 (55.4)	
Month 12	Good response	73 (19.5)	55 (20.1)	0.499
	Moderate response	66 (17.6)	49 (18.8)	
	Non-response	236 (62.9)	157 (60.2)	
Month > 12	Good response	54 (20.5)	28 (19.9)	0.998
	Moderate response	45 (17.1)	26 (18.4)	
	Non-response	164 (62.4)	87 (61.7)	

*All values are n (%). DAS28-based EULAR response: good response, ΔDAS28 > 1.2 and final DAS28 ≤ 3.2; moderate response, ΔDAS28 > 0.6 and ≤ 1.2 and final DAS28 ≤ 5.1 or ΔDAS28 > 1.2 and final DAS28 > 3.2; non-response, ΔDAS28 ≤ 0.6 or ΔDAS28 > 0.6 and ≤ 1.2 and final DAS28 > 5.1; ΔDAS28: mean improvement from baseline*.

### Assessment Outcomes

**Figure 2:**
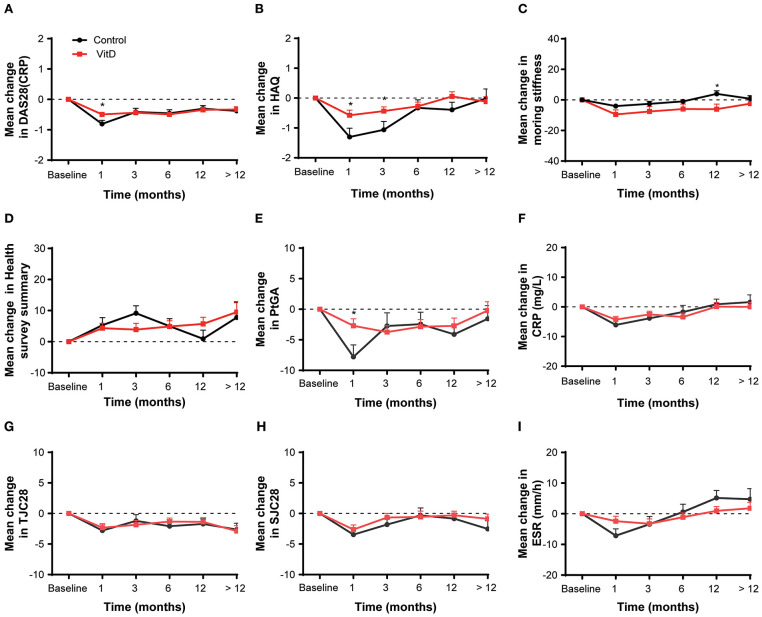
Mean change from baseline for all analyses in the overall patient cohort at months 1, 3, 6, 12, and > 12. **(A)** DAS28 (CRP), **(B)** HAQ, **(C)** Morning stiffness, **(D)** Health survey summary, **(E)** PtGA, **(F)** CRP, **(G)** TJC28, **(H)** SJC28, **(I)** ESR. DAS28, 28-joint disease activity score; HAQ, health assessment questionnaire; PtGA, patient global assessment; CRP, C-reactive protein; TJC28, tender 28-joint counts; SJC28, swollen 28-joint counts; ESR, erythrocyte sedimentation rate. *Indicates the differences of groups.

The secondary outcomes were the improvement of disease activity indexes in RA patients. Table 3 shows the mean clinical assessment values of all indicators at months 1, 3, 6, 12, and >12. The improvement in disease activity and the change trend are shown in Figure 2 and Table 4.

**Table 3:** Mean of clinical assessment outcomes at months 1, 3, 6, 12, and > 12.

**Characteristics**	**VitD group (*****N*** **=** **712)**					**Control group (*****N*** **=** **468)**				
	**1**	**3**	**6**	**12**	**> 12**	**1**	**3**	**6**	**12**	**> 12**
DAS28 (CRP)	2.75 ± 1.05	2.67 ± 1.15	2.60 ± 1.09	2.85 ± 1.25	2.76 ± 1.11	2.99 ± 1.17	3.12 ± 1.28	3.05 ± 1.30	3.19 ± 1.38	3.13 ± 1.17
HAQ	0.86 ± 2.09	0.89 ± 2.04	0.87 ± 2.37	1.32 ± 2.95	1.04 ± 2.69	1.25 ± 2.55	1.30 ± 2.34	1.62 ± 2.87	1.67 ± 2.98	1.94 ± 3.36
Morning stiffness (minutes)	5.94 ± 15.86	7.59 ± 21.87	7.27 ± 22.42	10.85 ± 31.25	9.20 ± 23.79	9.29 ± 20.23	10.88 ± 26.45	10.58 ± 24.34	13.00 ± 28.70	14.41 ± 32.40
Health survey summary	75.19 ± 15.71	73.31 ± 18.60	74.85 ± 17.56	73.81 ± 17.12	75.45 ± 17.49	70.69 ± 17.47	73.49 ± 17.45	70.91 ± 19.19	67.97 ± 19.99	63.62 ± 19.33
TJC28	3.80 ± 4.32	4.18 ± 5.18	4.16 ± 4.93	4.69 ± 5.67	3.83 ± 5.21	5.09 ± 5.69	5.68 ± 6.21	4.89 ± 4.58	5.70 ± 6.35	4.43 ± 5.78
SJC28	3.00 ± 3.50	4.13 ± 4.70	4.42 ± 5.23	4.01 ± 5.00	3.05 ± 3.59	4.05 ± 4.22	4.39 ± 4.55	4.68 ± 5.13	4.68 ± 5.51	4.36 ± 4.82
ESR (mm/h)	21.92 ± 21.74	19.90 ± 21.20	19.70 ± 20.11	20.56 ± 24.04	20.07 ± 23.15	15.49 ± 19.86	16.22 ± 21.48	19.98 ± 27.38	23.13 ± 26.26	20.65 ± 27.11
CRP (mg/L)	8.47 ± 16.94	8.71 ± 17.45	7.82 ± 17.58	11.00 ± 22.13	10.21 ± 20.61	8.95 ± 16.13	11.32 ± 18.69	12.92 ± 23.00	13.23 ± 23.13	13.36 ± 20.43
PtGA	42.51 ± 15.70	39.71 ± 16.83	39.92 ± 17.89	40.01 ± 18.41	40.81 ± 18.21	41.49 ± 16.73	42.97 ± 18.62	43.45 ± 20.10	41.55 ± 19.21	44.96 ± 16.38

*All values are n (%), mean ± SD. DAS28, 28-joint disease activity score; HAQ, health assessment questionnaire; TJC28, tender 28-joint counts; SJC28, swollen 28-joint counts; ESR, erythrocyte sedimentation rate; CRP, C-reaction protein; PtGA, patient global assessment*.

**Table 4:** Mean change from baseline at months 1,3, 6,12, and > 12.

**Characteristics**	**VitD group (*****N*** **=** **712)**					**Control group (*****N*** **=** **468)**				
	**1**	**3**	**6**	**12**	**> 12**	**1**	**3**	**6**	**12**	**> 12**
ΔDAS28	−0.49 ± 1.35	−0.44 ± 1.44	−0.49 ± 1.42	−0.34 ± 1.54	−0.33 ± 1.43	−0.81 ± 1.64	−0.42 ± 1.63	−0.46 ± 1.58	−0.31 ± 1.69	−0.38 ± 1.57
ΔHAQ	−0.57 ± 3.18	−0.44 ± 2.74	−0.28 ± 2.72	0.05 ± 3.04	−0.12 ± 2.70	−1.31 ± 4.06	−1.06 ± 3.74	−0.33 ± 3.73	−0.39 ± 4.06	−0.01 ± 3.68
ΔMorning stiffness	−4.02 ± 26.32	−2.60 ± 31.61	−0.95 ± 28.18	3.94 ± 35.92	0.77 ± 27.12	−9.47 ± 35.05	−7.57 ± 38.77	−5.94 ± 38.87	−6.01 ± 44.20	−2.40 ± 50.86
ΔHealth survey summary	4.34 ± 19.54	3.89 ± 23.63	4.89 ± 19.43	5.71 ± 19.07	9.52 ± 21.07	5.32 ± 21.87	9.14 ± 20.65	5.00 ± 19.64	0.93 ± 23.87	7.78 ± 15.11
ΔTJC28	−2.32 ± 6.25	−1.85 ± 7.87	−1.33 ± 6.47	−1.37 ± 8.28	−2.88 ± 8.24	−2.79 ± 9.85	−1.21 ± 9.18	−2.08 ± 9.03	−1.70 ± 8.93	−2.63 ± 7.91
ΔSJC28	−2.62 ± 6.43	−0.65 ± 5.77	−0.56 ± 7.31	−0.30 ± 6.16	−0.87 ± 5.18	−3.46 ± 7.61	−1.81 ± 6.58	−0.30 ± 8.28	−0.84 ± 7.20	−2.52 ± 6.80
ΔESR (mm/h)	−2.42 ± 28.42	−3.24 ± 28.79	−1.13 ± 25.92	0.94 ± 24.98	1.77 ± 29.37	−7.16 ± 27.49	−3.42 ± 28.78	0.58 ± 30.72	5.15 ± 34.69	4.76 ± 36.49
ΔCRP (mg/L)	−4.30 ± 20.21	−2.53 ± 20.62	−3.41 ± 24.48	0.08 ± 25.93	0.03 ± 24.06	−6.09 ± 23.05	−3.85 ± 24.05	−1.72 ± 28.51	0.88 ± 26.06	1.63 ± 27.07
ΔPtGA	−2.71 ± 21.13	−3.74 ± 20.57	−2.85 ± 22.33	−2.72 ± 24.75	−0.20 ± 22.97	−7.79 ± 25.35	−2.75 ± 26.48	−2.45 ± 25.47	−4.11 ± 25.68	−1.56 ± 24.48

*All values are n (%), mean ± SD. Δ: Mean change from baseline. DAS28, 28-joint disease activity score; TJC28, tender 28-joint counts; SJC28, swollen 28-joint counts; ESR, erythrocyte sedimentation rate; CRP, C-reaction protein; PtGA, patient global assessment; HAQ, health assessment questionnaire*.

At month 1, the VitD group did not provide more benefit for treatment RA than the control group. The improvement on DAS28 (CRP) (ΔDAS28 = −0.49 ± 1.35) for the VitD group was significantly lower than the control group (ΔDAS28 = −0.81 ± 1.64; *p* = 0.016). Meanwhile, the reduction of the HAQ for the VitD group (ΔHAQ = −0.57 ± 3.18) were significantly lower, compared with the control group (ΔHAQ = −1.30 ± 4.06; *p* = 0.021). The mean change of PtGA had significant difference between the VitD group and control group (ΔPtGA = −2.71 ± 21.13 vs. −7.79 ± 25.35; *p* = 0.017).

At month 3, the mean change in DAS28 (CRP) were −0.44 ± 1.44 for the VitD group and −0.42 ± 1.63 in the control group, without significant difference. For the HAQ, the improvement in the VitD group was lower than the control group (−0.44 ± 2.74 vs. −1.06 ± 3.74; *p* = 0.047). There were no differences for the other disease activity indicators between groups.

At months 6, 12, and >12, the mean changes of almost disease activity index between the two groups were similar.

### Subgroup Outcomes

**Figure 3:**
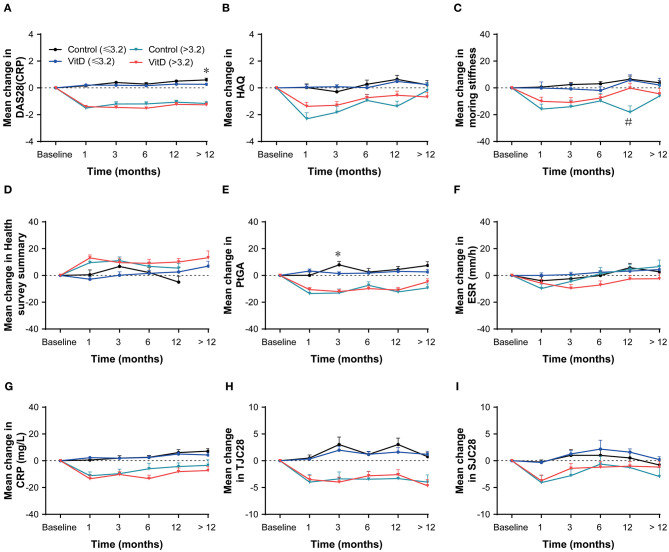
Subgroup analysis based on baseline DAS28 (CRP) at months 1, 3, 6, 12, and > 12. **(A)** DAS28 (CRP), **(B)** HAQ, **(C)** Morning stiffness, **(D)** Health survey summary, **(E)** PtGA, **(F)** ESR, **(G)** CRP, **(H)** TJC28, **(I)** SJC28. DAS28, 28-joint disease activity score; HAQ, health assessment questionnaire; PtGA, patient global assessment; ESR, erythrocyte sedimentation rate; CRP, C-reactive protein; TJC28, tender 28-joint counts; SJC28, swollen 28-joint counts. *Indicates the differences of baseline DAS28 ≤ 3.2 subgroup. ^#^Indicates the differences of baseline DAS28 > 3.2 subgroup.

After stratification according to the baseline DAS28 (CRP), the improvement of disease activity for the VitD group revealed a consistent tendency compared with control group in the both subgroups (Figure 3). It suggested that VitD supplementation did not bring treatment benefit than control group. In the baseline DAS28 (CRP) ≤ 3.2 subgroup, the analysis outcomes on most of disease activity index showed no differences between the VitD group and control group at each timepoint. Only the increasing of PtGA in VitD group was lower than control group at month 3 (*p* = 0.015), and at month > 12, and the increasing of DAS28 (CRP) was lower in the VitD group than the control group (*p* = 0.038). In the baseline DAS28 (CRP) > 3.2 subgroup, the outcomes demonstrated that the improvement of DAS28 (CRP) was similar at each timepoint between the two groups. As the same as above, the mean change for other parameters were similar between-groups.

**Figure 4:**
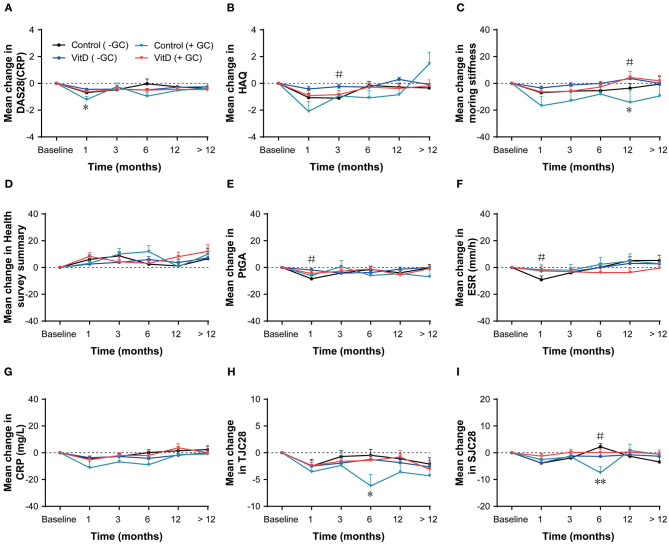
Subgroup analysis based on glucocorticoid (present/absent) at months 1, 3, 6, 12, nd > 12. **(A)** DAS28 (CRP), **(B)** HAQ, **(C)** Morning stiffness, **(D)** Health survey summary, **(E)** PtGA, **(F)** ESR, **(G)** CRP, **(H)** TJC28, **(I)** SJC28. GC, glucocorticoid; DAS28, 28-joint disease activity score; HAQ, health assessment questionnaire; PtGA, patient global assessment; ESR, erythrocyte sedimentation rate; CRP, C-reactive protein; TJC28, tender 28-joint counts; SJC28, swollen 28-joint counts. *Indicates the differences of glucocorticoid present subgroup (*p < 0.05; **p < 0.01). ^#^Indicates the differences of glucocorticoid absent subgroup.

Based on anti-inflammatory and immunosuppressive (24) benefits of glucocorticoids (GCs), they are usually used to therapy RA. The subgroup analysis was performed by GCs use (present/absent) and indicated that VitD supplementation did not impact the treatment (Figure 4). In the GCs present subgroup, only the mean change of DAS28 (CRP) at month 1 for the VitD group was lower than the control group (*p* = 0.036). Additionally, no differences were observed in the GCs absent subgroup at each timepoint.

**Figure 5:**
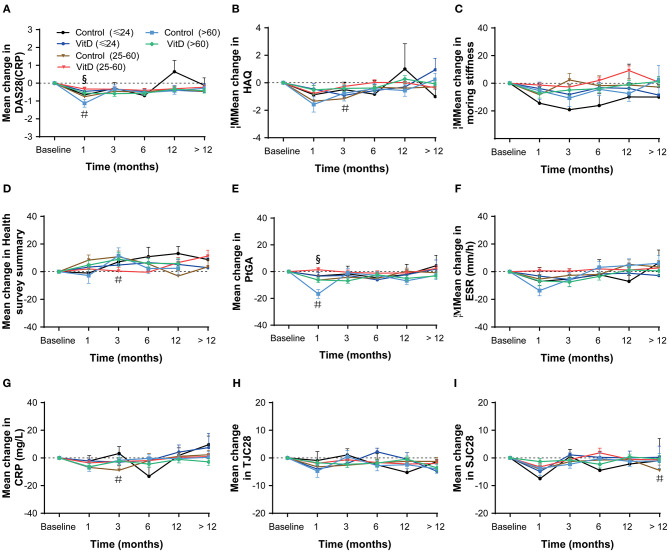
Subgroup analysis based on disease duration (≤ 24; > 24 and ≤ 60; > 60 months) at months 1, 3, 6, 12, and > 12. **(A)** DAS28 (CRP), **(B)** HAQ, **(C)** Morning stiffness, **(D)** Health survey summary, **(E)** PtGA, **(F)** ESR, **(G)** CRP, **(H)** TJC28, **(I)** SJC28. DAS28, 28-joint disease activity score; HAQ, health assessment questionnaire; PtGA, patient global assessment; ESR, erythrocyte sedimentation rate; CRP, C-reactive protein; TJC28, tender 28-joint counts; SJC28, swollen 28-joint counts. ^#^Indicates the differences of duration 25–60 subgroup. ^§^Indicates the differences of duration > 60 subgroup.

The disease duration subgroups analysis was performed (duration ≤ 24 months; > 24 and ≤ 60 months; > 60 months), and the results suggested that the VitD group did not revealed any advantage than the control group, as shown in Figure 5. The results of disease duration ≤ 24 months subgroup showed no differences for all variables between-groups at 1, 3, 6, 12, and > 12 months. In the duration > 24 and ≤ 60 months subgroup, at month 3, the significant lower mean change in the HAQ, the health survey summary and CRP, were shown for the VitD group than control group. The results in the RA > 60 months subgroup showed no differences for most of the disease activity parameters at each time point, except the improvement of DAS28 (CRP), which was lower in the VitD group than control group at month 1.

### Safety

The incidence of infections was generally comparable between the two groups, with no significant difference (*p* = 0.337) (Table 5). In total, 11 infection events were observed in the control group (8 cases of lung infection, 1 case of interstitial lung disease with infection, 1 case of urinary tract infection, and 1 case of infectious fever); and 21 infection events were observed in the VitD group (14 cases of lung infection, 1 case of interstitial lung disease with infection, 4 cases of bronchiectasis with infection, and 2 cases of anterior gastric ulcer with Helicobacter pylori infection).

**Table 5:** Summary of infections.

**Infections**	**VitD group**	**Control group**	***P***
Lung infection	14	8	
Bronchiectasis with infection	4	0	
Interstitial lung disease with infection	1	1	
Urinary tract infection	0	1	
Infectious fever	0	1	
Anterior gastric ulcer with helicobacter pylori infection	2	0	
Total	21	11	0.337

## Discussion

Because of the immunomodulatory effects of VitD, it provides opportunities to enhance the treatment of RA. In the present study, we primarily investigated whether VitD supplementation was associated with the response to antirheumatic treatment and improvement in disease activity for RA patients. We retrospectively analyzed 1,180 patients' medical records and found that VitD supplementation was not associated with an additional DAS28-based EULAR response to antirheumatic treatment at months 3, 6, 12, and > 12. The outcomes of disease activity indicators assessment showed a consistent change trend between the VitD group and the control group; a similar result was observed in the subgroup analyses. In summary, our study suggested that VitD supplementation did not produce an additional advantage for treating RA.

Until now, the evidence from prior randomized clinical trials (13–15) was not sufficient to determine the clinical benefits of VitD supplementation for RA treatment. However, it was confirmed that the serum VitD status was obviously increased by VitD supplementation in VitD-deficient and VitD-sufficient adults, which improved hypovitaminosis D in RA patients (16, 25). A previous double-blind study (26) reported that VitD supplementation showed no significant improvement in response to RA treatment, with a similar DAS 28 response in the VitD and placebo groups at month 3. Our outcomes were in line with those of the above study and demonstrated that VitD supplementation was not associated of the response to RA treatment at month 3. Subsequently, we assessed the response outcomes for longer periods, and the results still indicated no additional benefit for VitD supplementation at months 6, 12, or > 12. Our study evaluated the potential impact of VitD supplementation on the treatment of RA over a longer consecutive period.

An open-label interventional study (16) suggested that there was a significant improvement in DAS28 (CRP) after VitD (cholecalciferol) supplementation for more than 3 months in patients with active RA and hypovitaminosis D. The authors mainly contrasted the change in DAS28 (CRP) from baseline 3.68 ± 0.93 to 3.08 ± 1.11 at month 3 in patients who received VitD supplementation, without data from patients who did not receive VitD. In our study, the mean change in the DAS28 (CRP) from baseline 3.24 ± 1.42 to 2.67 ± 1.15 (*p* < 0.001) in the VitD group, as compared to that in the control group (from 3.64 ± 1.51 to 3.12 ± 1.28, *p* < 0.001). At month 3, significant improvements in the DAS28 (CRP) were reported in both groups. Thus, we compared the mean change in the DAS28 (CRP) (ΔDAS28 (CRP)) between the two groups and did not observe significant difference. We reported a different outcome: VitD supplementation did not contribute to additional improvement of DAS28 (CRP) in RA patients. This may be due to our larger sample size and different RA patient backgrounds (VitD deficient and sufficient). Moreover, the VitD supplementation schemes (the dose and duration of supplementation VitD) were different. In the open-label study (16), patients were treated with 60,000 IU/week for 6 weeks, followed by 60,000 IU/month for 3 months. Furthermore, we need to evaluate the different doses of VitD supplementation that impact the treatment of RA to enhance our understanding of the potential role of VitD.

A prior clinical trial (27) suggested that high doses of VitD (cholecalciferol, 100,000 IU) supplementation led to a statistically significant improvement in the HAQ compared with the placebo-controlled group among RA patients with VitD deficiency (serum VitD levels <30 ng/mL) at month 6. This was inconsistent with our outcomes, which showed no significant difference when comparing the mean change in the HAQ between the two groups at month 6. This difference may be due to different patient baseline VitD levels and the doses of VitD supplementation. Interestingly, our reassessments revealed that VitD supplementation had an inverse association with improved HAQ, that is, it was a risk factor in RA patients at month 1 and month 3. This was in agreement with a study (28), that suggested that multiple high VitD exposures might increase RA incidence.

A subgroup analysis was performed based on the baseline disease activity (DAS28 (CRP)) to investigate whether VitD supplementation utility or antirheumatic treatment was associated with baseline disease activity. All patients were divided into two subgroups: the DAS28 (CRP) ≤ 3.2 subset, which included remission and low disease activity patients; and the DAS28 (CRP) >3.2 subset, which included moderate and high activity patients (29). According to our assessment, the efficacy of antirheumatic treatment was different between the two subsets, with slightly increasing DAS28 (CRP) in the DAS28 (CRP) ≤ 3.2 subset, and obviously decreasing DAS28 (CRP) in the DAS28 (CRP) > 3.2 subset. This indicated that antirheumatic efficacy was related to baseline disease activity. However, we found that VitD supplementation did not affect the efficacy of antirheumatic treatment in the DAS28 (CRP) ≤ 3.2 subset, with no difference between the VitD group and control group. A similar result was revealed from the other disease activity indexes. Taken together, these results demonstrated that VitD supplementation did not affect the efficacy of antirheumatic treatment.

A few animal studies (30, 31) have reported a possible anti-inflammatory role of GCs in RA. GCs was widely used to treat RA (32). In the subgroup analysis based on baseline GCs use (present/absent), the tendency of change for all disease activity indexes was consistent between the VitD group and control group in both subsets. This might indicate that the role of VitD is independent of GCs usage.

Infections were considered to be important risk factors related to the progression of RA. A prior study suggested that the overall infection rate increased significantly during RA onset, and the antibacterial defense mechanism was defective (33). Recently, authors reported that VitD supplementation effectively reduced the incidence of acute cellular rejection and infection (34, 35), upper respiratory infection (36, 37) and acute respiratory infections (36, 38). However, we obtained different results compared with the above studies. Our study demonstrated that VitD supplementation did not reduce the rate of all causes of infections, and no difference between the VitD and control groups were observed (*p* > 0.05). Most of the infection events were lower respiratory tract infections, including 22 cases of lung infection, 4 cases of bronchiectasis with infection, and 2 cases of interstitial lung disease with infection.

A few limitations were recognized in our study. First, it was a single-center and retrospective study. The study population was unbalanced in terms of disease duration, disease activity and oral glucocorticoids at baseline. In order to minimize potential data bias, we used the Δ value (change from baseline) of disease activity indicators for contrasting the anti-rheumatic efficacy, further, we also performed subgroup analysis based on baseline DAS28, glucocorticoids (present/absent) and disease duration. Therefore, it is necessary to design prospective randomized controlled studies to further verify our results. Additionally, in our single-center study, the generalizability of these results may be limited due to the relatively single study population and fewer types of VitD drugs. In the later, we plan to design a multi-center study to further confirm the role of VitD on RA in wider population. Second, the VitD supplementation schemes were inconsistent. Third, most patients did not have baseline serum levels of 25OHD to determine their original VitD status. Finally, a number of missing data points led to reduced objective accuracy of the final evaluation results. In the future, we expect to design multicenter, prospective studies with large sample sizes to enhance the evidence for these conclusions.

## Conclusion

In conclusion, VitD supplementation did not provide a statistically significant improvement in treatment response or disease activity for RA patients who stably continued anti-rheumatic treatment with csDMARDs. The role of VitD supplementation in RA needs further research.

## Data Availability Statement

All datasets presented in this study are included in the article/supplementary material.

## Ethics Statement

The studies involving human participants were reviewed and approved by Medical ethics committees of Dazhou Central Hospital. Written informed consent for participation was not required for this study in accordance with the national legislation and the institutional requirements.

## Author Contributions

FanxZ, JY, JW, and SL contributed conception and design of the study. All authors organized the database. SL performed the statistical analysis. FanxZ and SL wrote the draft of the manuscript. All authors contributed to manuscript revision, read, and approved the submitted version.

## Conflict of Interest

The authors declare that the research was conducted in the absence of any commercial or financial relationships that could be construed as a potential conflict of interest.
